# SiRNA-Induced Mutation in HIV-1 Polypurine Tract Region and Its Influence on Viral Fitness

**DOI:** 10.1371/journal.pone.0122953

**Published:** 2015-04-10

**Authors:** Jason W. Rausch, Meijuan Tian, Yuejin Li, Lora Angelova, Bernard S. Bagaya, Kendall C. Krebs, Feng Qian, Chuanwu Zhu, Eric J. Arts, Stuart F. J. Le Grice, Yong Gao

**Affiliations:** 1 HIV Drug Resistance Program, Frederick National Laboratory for Cancer Research, Frederick, Maryland, United States of America; 2 Division of Infectious Diseases, Department of Medicine, Case Western Reserve University, Cleveland, Ohio, United States of America; 3 Department of Molecular Biology and Microbiology, Case Western Reserve University, Cleveland, Ohio, United States of America; 4 Suzhou Fifth People’s Hospital, Suzhou, Jiangsu, China; 5 Department of Microbiology and Immunology, Western University, London, Ontario, Canada; Institut Pasteur, FRANCE

## Abstract

Converting single-stranded viral RNA into double stranded DNA for integration is an essential step in HIV-1 replication. Initial polymerization of minus-strand DNA is primed from a host derived tRNA, whereas subsequent plus-strand synthesis requires viral primers derived from the 3′ and central polypurine tracts (3′ and cPPTs). The 5′ and 3′ termini of these conserved RNA sequence elements are precisely cleaved by RT-associated RNase H to generate specific primers that are used to initiate plus-strand DNA synthesis. In this study, siRNA wad used to produce a replicative HIV-1 variant contained G(-1)A and T(-16)A substitutions within/adjacent to the 3′PPT sequence. Introducing either or both mutations into the 3′PPT region or only the G(-1)A substitution in the cPPT region of NL4-3 produced infectious virus with decreased fitness relative to the wild-type virus. In contrast, introducing the T(-16)A or both mutations into the cPPT rendered the virus(es) incapable of replication, most likely due to the F185L integrase mutation produced by this nucleotide substitution. Finally, the effects of G(-1)A and T(-16)A mutations on cleavage of the 3′PPT were examined using an in vitro RNase H cleavage assay. Substrate containing both mutations was mis-cleaved to a greater extent than either wild-type substrate or substrate containing the T(-16)A mutation alone, which is consistent with the observed effects of the equivalent nucleotide substitutions on the replication fitness of NL4-3 virus. In conclusion, siRNA targeting of the HIV-1 3′PPT region can substantially suppress virus replication, and this selective pressure can be used to generate infectious virus containing mutations within or near the HIV-1 PPT. Moreover, in-depth analysis of the resistance mutations demonstrates that although virus containing a G(-1)A mutation within the 3′PPT is capable of replication, this nucleotide substitution shifts the 3′-terminal cleavage site in the 3′PPT by one nucleotide (nt) and significantly reduces viral fitness.

## Introduction

Converting single-stranded viral RNA into double stranded DNA for integration is an essential step in HIV-1 replication. This process is mediated by reverse transcriptase (RT), a multifunctional enzyme with both DNA polymerase and ribonuclease H (RNase H) activities [1–3]. Initial polymerization of minus-strand DNA is primed by a host-derived tRNA, whereas subsequent plus-strand synthesis requires viral primers produced by RNase H cleavage of the 3′ and central polypurine tracts (3′ and cPPTs) [4].

PPTs are essential, conserved sequence elements found within the RNA genomes of all retroviruses. While most HIV RNA is ultimately degraded by reverse transcriptase (RT)-associated ribonuclease H (RNase H), PPTs are relatively resistant to RNA degradation and remain hybridized to nascent minus-strand DNA in order to serve as plus-strand primers [4]. All retroviruses contain at least one PPT sequence near the 3′ end of the viral genome. This element, designated the 3′PPT, universally serves as a plus-strand initiation site and marks the 5′ end of U3. Most lentiviruses also contain a second PPT, central PPT (cPPT) located within the integrase gene near the center of the genome and having a sequence identical (or nearly identical) to that of the 3′PPT. Plus-strand synthesis initiates from the cPPT as well, which is the reason why the plus-strand in HIV pre-integrative DNA is discontinuous [5, 6]. In HIV-1, the ends of the 3′ and central PPT sequences are precisely cleaved to generate mainly 17 or 19 nt plus-strand primers [7, 8]. RT selectively utilizes these primers to initiate (+) strand DNA synthesis, after which at least the 3′PPT is removed by RNase H-mediated cleavage. 3′PPT processing ultimately serves to define the extreme 5′ terminus of the viral DNA before it is integrated into the host genome [9–13]. Consequently, precision in plus-strand initiation and PPT removal is crucial for viral replication [14, 15].

HIV-1 RT has been evolutionarily selected to specifically cleave the PPTs between the 3′ terminal G and A ribonucleotides while leaving the remainder of the purine-rich sequence element intact. Although the structural basis for this cleavage specificity remains poorly understood, several lines of evidence suggest that RNA/DNA hybrid harboring the PPT sequence contains unique structural features that may be specifically recognized by HIV-1 RT. For instance, NMR and other structure analyses of PPT-containing RNA/DNA and DNA/DNA duplexes have revealed that the A-tracts in the PPT have a narrow minor groove and unusual C2-endo sugar conformation [16–20]. Distorted A-tracts cannot be the only structural determinant for specific PPT recognition by HIV-1 RT however, as several mutational studies indicate that an intact 3′ terminal stretch of G nucleotides is more important for PPT function [14, 20–25]. Unfortunately, many of these studies were conducted exclusively *in vitro*, so the *in vivo* relevance of the findings cannot be directly confirmed. Moreover, most prior mutational studies, even those conducted using cell culture, examined the effects of arbitrarily generated mutations and revealed little regarding the flexibility of the PPT sequences when exposed to selective pressure.

Structure-function studies on HIV-1 replication can often be guided by natural sequence variation during viral evolution or by escape mutations that arise due to drug or immune pressure. Remarkably, the sequences of both the central and 3′PPT are nearly invariant in all HIV-1 groups and subtypes. Even the PPTs of HIV-1 and HIV-2 are almost identical despite the two lentivirus types sharing less than 60% sequence identity overall. The HIV-1 central PPT is located in the integrase coding sequence (nt position 4785–4799, HXB2 numbering) and is part of the KY9 CTL epitope restricted by B27 HLA alleles [26]. Even though this epitope is recognized by some CD8+ T cells in B27+ patients, there have been no reports of escape or sequence variation aside from purine-to-purine substitutions that maintain coding sequence (http://www.hiv.lanl.gov). Moreover, mutational studies of the cPPT demonstrate that modifying the cPPT sequence typically results in loss of viral infectivity or significant delay of virus replication [6, 27, 28].

In the present study, we use siRNA to exert selective pressure against the HIV-1 3′PPT as a means of screening for naturally escaped replication competent 3’PPT mutant(s) and objectively determining the degree to which this highly conserved sequence element is genetically flexible. Specifically, primary HIV-1 isolates and a laboratory strain were serially passaged in the presence of escalating concentrations of siRNA targeting the 3′PPT. Of the three siRNAs tested, one consistently suppressed replication of all strains tested when present at high concentration. However, an emergent variant of the v120-A strain (a subtype A strain from Uganda) was identified and found to have two nucleotide substitution mutations within the 3′PPT. By transplanting these mutations individually and in combination into the central and/or 3′PPT sequence of NL4-3, we were able to determine not only the effects of these mutations on PPT function, but also the relative importance of the two PPTs in virus replication. SiRNA resistance and relative fitness levels were evaluated for all mutant variants. Overall, the level of siRNA resistance in mutant HIV-1 isolates was modest and only conferred by introducing mutations into the 3′PPT. The costs of even this moderate degree of resistance were substantial, however, as introducing both mutations, or even the G(-1)A mutation alone into the 3′PPT of NL4-3 significantly reduced viral fitness relative to wild type. This may be the result of altered RNase H processing of the mutant PPT primer, as in vitro RNase H cleavage analysis indicated that mutant 3′PPTs are cleaved internally to a greater extent than is observed with the wild type substrate. Altered PPT processing could in turn reduce the efficiency of plus-strand priming and primer removal while also adversely affecting integration of HIV-1 dsDNA into the genome of the infected cell.

## Materials and Methods

### Ethics Statement

Peripheral blood mononuclear cells (PBMCs) were obtained from HIV patients and HIV-1 seronegative donors at Uganda. The sample collection was approved by Institutional Review Board (IRB) in the Joint Clinical Research Center (JCRC), Uganda and Case Western Reserve University (CWRU). Informed written consent was obtained from each participant.

### Cell culture

PBMCs were isolated from HIV-1 seronegative donors by Ficoll-Paque density centrifugation and cultured in RPMI-1640 medium containing 10% fetal bovine serum (FBS) as described [29]. U87.CD4.CXCR4 cell line was obtained from the AIDS Research and Reference Reagent Program and grown in Dulbecco’s modified Eagle’s medium (DMEM, Cellgro) supplemented with 15% FBS, penicillin and streptomycin, puromycin (1 μg/ml) and G418 sulfate (1 mg/ml). 293T cells were obtained from the American Type Culture Collection and grown in DMEM supplemented with 10% FBS, penicillin and streptomycin. All cells were grown at 37°C in 5% CO_2_.

### Viruses

The CXCR4-tropic, primary HIV-1 isolates v120-A (clade A) and v126-D (clade D) have been described previously [29]. The viruses were propagated by co-culturing PBMCs derived from the patients and from healthy donors. Tissue culture dose for 50% infectivity (TCID_50_) were determined using the Reed-Munch assay as previously described [30]. Titers are expressed as infectious units per milliliter. NL4-3, a laboratory adaptive virus [31], was used as a reference virus and a backbone for generation of various PPT mutants.

### siRNA preparation

Twenty-one-nucleotide dsRNAs were chemically synthesized as 2′ bis (acetoxyethoxy)-methyl ether-protected, desalted and duplexed oligonucleotides by Dharmacon (Lafayette, Colo.). 4 siRNAs were designed according to the manufacturer's recommendations. siRNA-PPT1, siRNA-PPT2, siRNA-PPT3, and siRNA-PPTmu were specifically designed to target HIV-1 3′PPT region based on NL4-3 sequence.

### Detection of siRNA inhibition on HIV-1 replication

**Fig 1:**
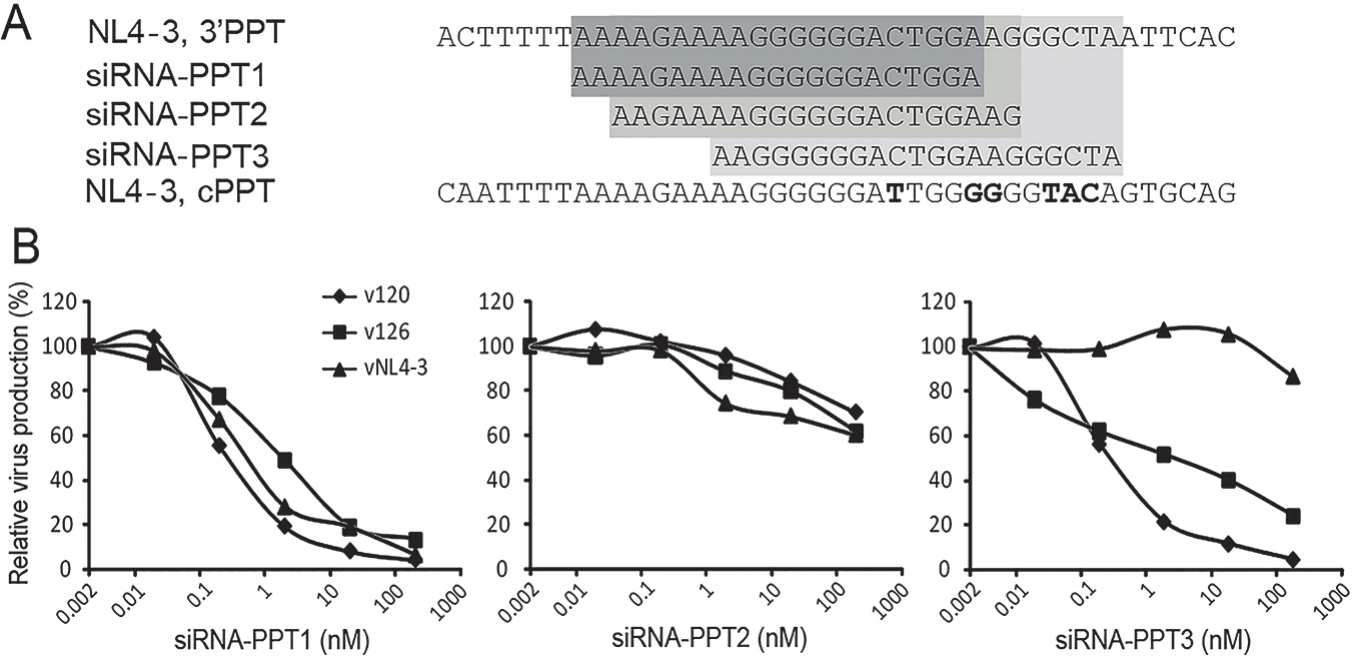
Design of siRNA-ppts and measuring their inhibition of HIV-1 replication. (A) Three siRNAs were designed to target the HIV-1 3′PPT region: siRNA-PPT1, siRNA-PPT2 and siRNA-PPT3 contain sequence matching nt9069-9089, nt9071-9091, and nt9076-9096 of HXB2, respectively. The sense, passenger strands of the respective siRNA duplexes are indicated and aligned with the corresponding 3’PPT and flanking sequences of NL4-3. Importantly, the targeted sequences within NL4-3, v120 and v126 virus strains are identical. (B) The efficiency of siRNA-PPT inhibition of HIV replication was monitored by RT activity assay.

U87.CD4.CXCR4 cells were plated in 24-well plates (1.0×10^5^/well), and 24 hours later, transfected with different siRNA using lipofectamine2000 (Invitrogen) at various concentrations according to manufacturer's instructions (Fig 1). The cells were then infected by v120-A (wild-type or mutant), v126-D or NL4-3 (primary or chimeric) at 0.1 MOI 16 h post-transfection of siRNAs. Virus was removed 5 hours post-infection at which point cells were washed with PBS 3 times and refilled with fresh media. Supernatant and/or cells were collected at different time points for further detections. The infected cells without siRNA treatment were used as a control. Reverse transcriptase (RT) activity in the supernatant was monitored to determine relative virus production through RT assay [32]. Briefly, supernatant samples (10 μl) clarified of cell debris by centrifugation at 2,500 × *g* for 5 min were added to 96-well plates along with 25 μl of RT master mix [50 mM Tris-HCl (pH 7.8), 75 mM KCl, 2 mM dithiothreitol, 5 mM MgCl_2_, 5 μg/ml poly(rA) poly(dT), 0.5% (vol/vol) NP-40, 1 μl/ml of fresh 10 mCi/ml [α-^32^P]-dTTP. After incubation at 37°C overnight, 10 μl of the RT reaction mixtures was blotted onto a DEAE filtermat (Wallac Oy, Turku, Finland), washed five times with SSC (0.15 M NaCl, 0.015 M sodium citrate), rinsed in 80% ethanol, and dried. Radioactivity (counts per minute) from each well on the dried filters was measured with a Matrix 96 direct beta counter (Packard, Meriden, Conn.). Incorporation of [α-^32^P]-dTTP by HIV-1 RT is a relative measure of RT activity and virus in the supernatant.

### Construction of 3′PPT and/or cPPT mutants

To construct the 3′PPT and cPPT mutants, yeast homologous recombination [33–35] was employed to generate pREC_nfl_NL4-3_Δ3′PPT/URA3 and pREC_nfl_NL4-3_ ΔcPPT/URA3, i.e. NL4-3 plasmids where a sequence encompassing the PPT was replaced by URA3. The same HIV-1 region replaced by URA3 was then PCR-amplified to generate fragment harboring the mutated 3′PPT or cPPT. These PCR-amplified fragment were then recombined/cloned into pREC_nfl_NL4-3_Δ3′PPT/URA3 and pREC_nfl_NL4-3_ ΔcPPT/URA3 to produce pREC_nfl_NL4-3_Δ3′PPT (3′PPT_G(-1)A_, 3′PPT_T(-16)A_, 3′PPT_G(-1)A/T(-16)A_), or pREC_nfl_NL4-3_ ΔcPPT (cPPT _G(-1)A_, cPPT_T(-16)A_, and cPPT_G(-1)A/T(-16)A_) (S1 Fig). The vectors of pREC_nfl_NL4-3_Δ3′PPT_ΔcPPT (cPPT _G(-1)A_/Δ3′PPT_G(-1)A/T(-16)A_) containing mutations in both of cPPT and 3′PPT were constructed using the same procedure. Briefly, The Saccharomyces cerevisiae yeast MYA-906 (genotype MATα-ade6-can1-his3-leu2-trp1-URA3) was grown in 5 ml YEPD media overnight with shaking at 30°C. 12–16 hours later, 0.5 ml of the yeast culture was inoculated into 50 ml YEPD media and grown for 4–6 hours to attain an OD600 = 1.0. The yeast was washed in 1 ml of sterile water and resuspended in 1 ml of fresh 1x LiAc/TE solution in water [LiAc (100 mM pH 7.5) and TE (10 mM Tris-Cl pH 7.5 and 1 mM EDTA pH 8.0)] to induce competence and then chilled on ice. The PCR insert (3μg) and the linearized corresponding plasmid (1μg) were then co-transformed along with 50 μg of denatured salmon sperm carrier DNA (BD Biosciences/Clontech, Palo Alto, CA) into yeast using the lithium acetate-PEG. The mixture was incubated on a shaking platform at 30°C for 30 minutes followed by a 15 minute heat shock at 42° and then plated on complete supplement media (CSM)-LEU+5FOA agar plates in which only the yeast containing successful replacement of URA by certain HIV sequence can grow. Plasmids were recovered from yeast clones using a mixture of mechanical glass bead disruption and phenol-chloroform-isoamyl alcohol (25:24:1) extraction, and 4μL of the crude yeast preparation was transformed into STBL4 E.coli (Invitrogen). The produced plasmids were finally extracted from STBL4 E. coli cells.

Each of the pREC_nfl_NL4-3 vectors harboring the PPT mutations were then co-transfected into 293T along with the complementing vector, pCMV_cplt using Effectene Transfection Reagent (QIAGEN). Virus-containing supernatant from the transfected 293T cells was harvested 48 hours post-transfection and used to infect U87.CD4.CXCR4 cells. RT activity (i.e. HIV-1 propagation) was measured in the cell-free supernatants of U87.CD4.CXCR4 cells at 3, 5, 7, 10, and 15 days post-infection.

### HIV-1 fitness estimates from growth competition infections and heteroduplex tracking assays (HTAs)

All infectious HIV-1 variants harboring mutated 3′PPT and/or cPPT were used to singly or dually infect U87.CD4.CXCR4 cells together with a NL4-3 reference virus containing wild-type PPTs and synonymous nucleotide substitutions in vif (designated vifB). This vifB tag was used to distinguish reference virus from NL4-3 clones harboring wild-type vif (vifA) [36]. Viruses were added alone or in pairs to the cells at a multiplicity of infection of 0.001 infectious unit/cell in a 24-well plate (1×10^5^ cells/well). After 5 hours of incubation at 37°C with 5% CO_2_, cells were washed three times with phosphate-buffered saline and then added back fresh complete medium. All mono- and dual-infection experiments were performed in triplicate. Uninfected cultures were used as HIV negative controls. Cell-free supernatants were assayed for RT activity at 3, 5, 7, and 10 days post-infection, and when the RT activity of the culture became positive (2000 cpm/ml or higher), cells were harvested for investigation of the viral fitness through heteroduplex tracking assays (HTAs) as described below.

In HTA assays, the HIV-1 vif gene was first PCR amplified from each dual- and mono-infection with primers VifFWD and VifNestRev as previously described [37]. A shorter vif fragment was also PCR amplified from pNL4-3vifA vector for use as a DNA probe with primers vifA-HTA and VifNestRev. For the latter amplification, primer Vif-HTA was radiolabeled with T4 polynucleotide kinase and 2 μCi of [^32^P]ATP. Radiolabeled PCR-amplified probes were separated on 1% agarose gels and purified using a QIAquick gel extraction kit (QIAGEN). HTA reaction mixtures containing DNA annealing buffer (100 mM NaCl, 10 mM Tris-HCl [pH 7.8], 2 mM EDTA), 10 μl of unlabeled PCR-amplified DNA from the competition culture, and approximately 0.1 pmol of radioactive probe DNA were denatured at 95°C for 3 min and annealed at 37°C for 5 min. After 30 min on ice, the DNA heteroduplexes were resolved on 6% nondenaturing polyacrylamide gels (acrylamide-bisacrylamide, 30:0.8) in Tris-borate-EDTA buffer for 4 h at 200 V. Gels were dried and exposed to X-ray film (Eastman Kodak Co., Rochester, N.Y.). Heteroduplexes representing the production of each isolate in a dual infection were quantified with a Bio-Rad PhosphorImager.

Relative fitness values (w) were obtained by calculating the fractional abundance of a given isolate following a dual infection (f_0_ = heteroduplex_isolate_/heteroduplex_total_) divided by the initial proportion of that isolate in the inoculum (i_0_, generally equal to 0.5); i.e., where w = f_0_/i_0_. The ratio between the relative fitness values of any two HIV-1 variants in a competition experiment is calculated as W_D_ = w_M_/w_L_ [29], where w_M_ and w_L_ correspond to the relative fitness values of the more fit and less fit viruses, respectively. It is important to note that the W_D_ metric is only useful when the isolates being compared have been competed against a common third isolate in separate HTA competition experiments.

### In vitro PPT RNA/DNA hybrids cleavage assay

As described previously [24, 38], four 5′ ^32^P-end-labeled 29-nt RNA oligonucleotides containing variations of the v120 3’ PPT and flanking sequences were annealed to complementary 39-nt DNAs such that the there is a 5 nt DNA overhang at either end. Annealing was performed by heating the mixture to 90°C and slow cooling to 4°C in 10 mm Tris/HCl (pH 7.6), 25 mm NaCl. RNA sequences within the hybrid substrates are as follows: WT—5′-32P-uuuuAAAAGAAAAGGGGGGactggatggg (the wild-type sequence); double mutant—uuu**A**AAAAGAAAAGGGGG**A**actggatggg (contains both the U(-16)A and G(-1)A substitutions found in the siRNA breakthrough virus); single mutant U(-1)A—uuu**A**AAAAGAAAAGGGGGGactggatggg; and single mutant G(-1)A—uuuuAAAAGAAAAGGGGG**A**actggatggg. These hybrids (final concentration 50 nM) were subjected to RNase H-mediated cleavage by HIV-1 RT (10nM) in 10 mM Tris-HCl (pH 8.0), 80 mM NaCl, and 6 mM MgCl2 at 37°C. Reactions were terminated at 0, 1, 3, 10, or 30 min by mixing with urea-based gel-loading buffer. Cleavage products were fractionated by high-voltage electrophoresis through denaturing PAGE and visualized by phosphorimaging.

## Results

### Screening for siRNAs effective in targeting the HIV-1 3′PPT region

Three different siRNAs were designed to target the HIV-1 3′PPT region (Fig 1A). SiRNA-PPT1, siRNA-PPT2 and siRNA-PPT3 target the positions nt9069-9089 (HXB2 numbering), nt9071-9091, and nt9076-9096, respectively. To determine the inhibitory efficacy of these three siRNAs on virus replication, U87.CD4.CXCR4 cells were transfected with different concentrations of each siRNA then infected with different HIV-1 viruses. Of the siRNAs tested, siRNA-PPT1 was the most potent, inhibiting the v120-A, v126-D and vNL4-3 HIV-1 strains with IC_50_ values of 0.25nM, 1.6nM and 0.41nM, respectively. SiRNA-PPT3 inhibited virus v120-A (IC_50_ = 0.29nM) and v126-D (IC_50_ = 3.2nM) replication but not vNL4-3. SiRNA-PPT2 was designed to target a sequence encompassing the 3′-terminal 14 nt of the 3′PPT, including the G-tract, as well as 7 nt immediately downstream (Fig 1A). However, this siRNA had no inhibitory activity against any of the three HIV-1 strains (Fig 1B).

### Selecting for siRNA-PPT resistant viruses

SiRNA-PPT1 and PPT3 were used to select for resistant HIV-1 isolates by escalating the dose of siRNA with each virus passage. These siRNAs were not developed as part of a therapeutic study but rather to induce the virus to acquire resistance mutations within the PPT while maintaining PPT function. We expected that this directed approach for analyzing PPT function would yield more functionally relevant results than random mutagenesis.

We have previously reported that HIV-1 specific siRNAs maintained efficient inhibition of virus replication for 5 days, with virus rebound typically observed by day 8 [39]. Similar inhibition kinetics was observed with the siRNA-PPTs used here. Therefore, to reduce outgrowth of wild-type virus, virus supernatants were harvested from siRNA treated cells on day 5 then added to U87.CD4.CXCR4 cells pre-treated with a 2- to 3-fold higher siRNA concentration. This process was repeated sequentially throughout the range of siRNA concentration tested (0.2 nM to 10 nM). Supernatant was collected for detection of virus production by RT assay, and cellular DNA was PCR amplified and sequenced through the central and 3′PPT regions.

**Fig 2:**
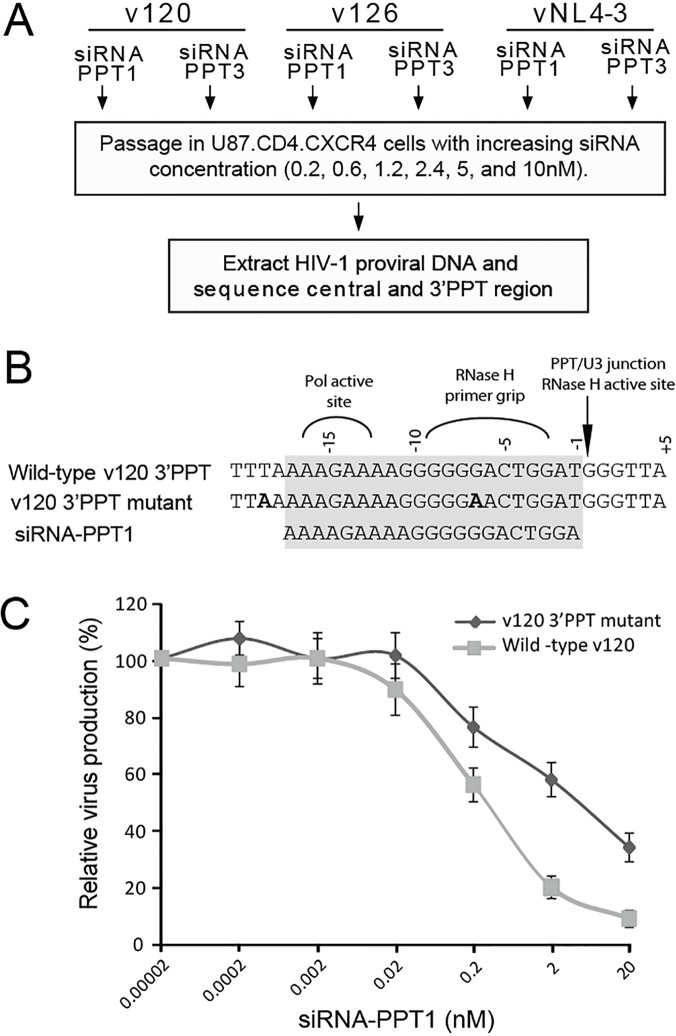
Generation of replication-competent PPT mutants through siRNA treatment. (A) Schematic illustrating the process of generating PPT mutants through siRNA treatment; (B) Mutated PPT in the emergent v120-A strain together with the wild-type 3′PPT and siRNA-PPT1 sequences; (C) Inhibitory efficiency of siRNA-PPT1 on wild-type and mutated v120-A.

After six rounds of siRNA treatment, v126-D and vNL4-3 replication was still effectively inhibited, suggesting that the genetic and/or fitness barrier to siRNA-PPT1 resistance is quite high. However, the v120-A virus evolved two mutations within or near the 3′PPT after siRNA-PPT1 exposure, indicating that perfect conservation of the purine rich element is not essential for virus survival. One mutation, G(-1)A (nt9083) was located within the siRNA-PPT1 target region, and the other, T(-16)A (nt9068) was found immediately upstream of the 3′PPT and siRNA-PPT1 target region (Fig 2B). Sequencing of regions adjacent to the central and 3′PPTs of the v120-A genome revealed no other nucleotide substitutions. We then compared the susceptibility of v120-A mutant (v120-A _PPT1_) and wild-type viruses to the siRNAs and found that the v120-A _PPT1_ mutant had developed resistance to siRNA-PPT1. The IC50 of v120-A _PPT1_ against siRNA-PPT1 was 4.1nM, a value 16.4 times higher than was measured for wild-type v120-A (Fig 2C).

### Generation of HIV-1 clones with mutations in the 3′PPT and/or cPPT

Based upon previous mutational analysis of PPT function [25], we suspected that the G(-1)A mutation in the v120-A 3′PPT G tract was responsible for conferring resistance to siRNA-PPT1, while the T(-16)A mutation only performed the compensatory function of maintaining the suitability of the PPT region as a primer for viral plus-strand DNA synthesis. To test this hypothesis, we introduced G(-1)A, T(-16)A or both mutations into the 3′PPT and/or cPPT regions in an NL4-3 backbone using a yeast-based recombination/cloning system for HIV-1 (S1 Fig) [33]. Six viruses were constructed as described in Materials and Methods harboring the G(-1)A, T(-16)A, or G(-1)A/T(-16)A substitutions in the 3′PPT or cPPT (i.e. 3′PPT_G(-1)A_, 3′PPT_T(-16)A_ and 3′PPT_G(-1)A/T(-16)A_, or cPPT _G(-1)A_, cPPT_T(-16)A_ and cPPT_G(-1)A/T(-16)A_, respectively). A seventh variant harboring the G(-1)A mutation in both the central and 3′PPT was also constructed (cPPT_G(-1)A_/3′PPT_G(-1)A_). 293T cells were transfected with the mutant NL4-3 vectors (e.g. pREC_nfl_HIV-1_NL4-3__3′PPT_G(-1)A_ or 3′PPT_G(-1)A_) and the complementing vector produced virus particles with both RT activity and p24 content [33].

**Fig 3:**
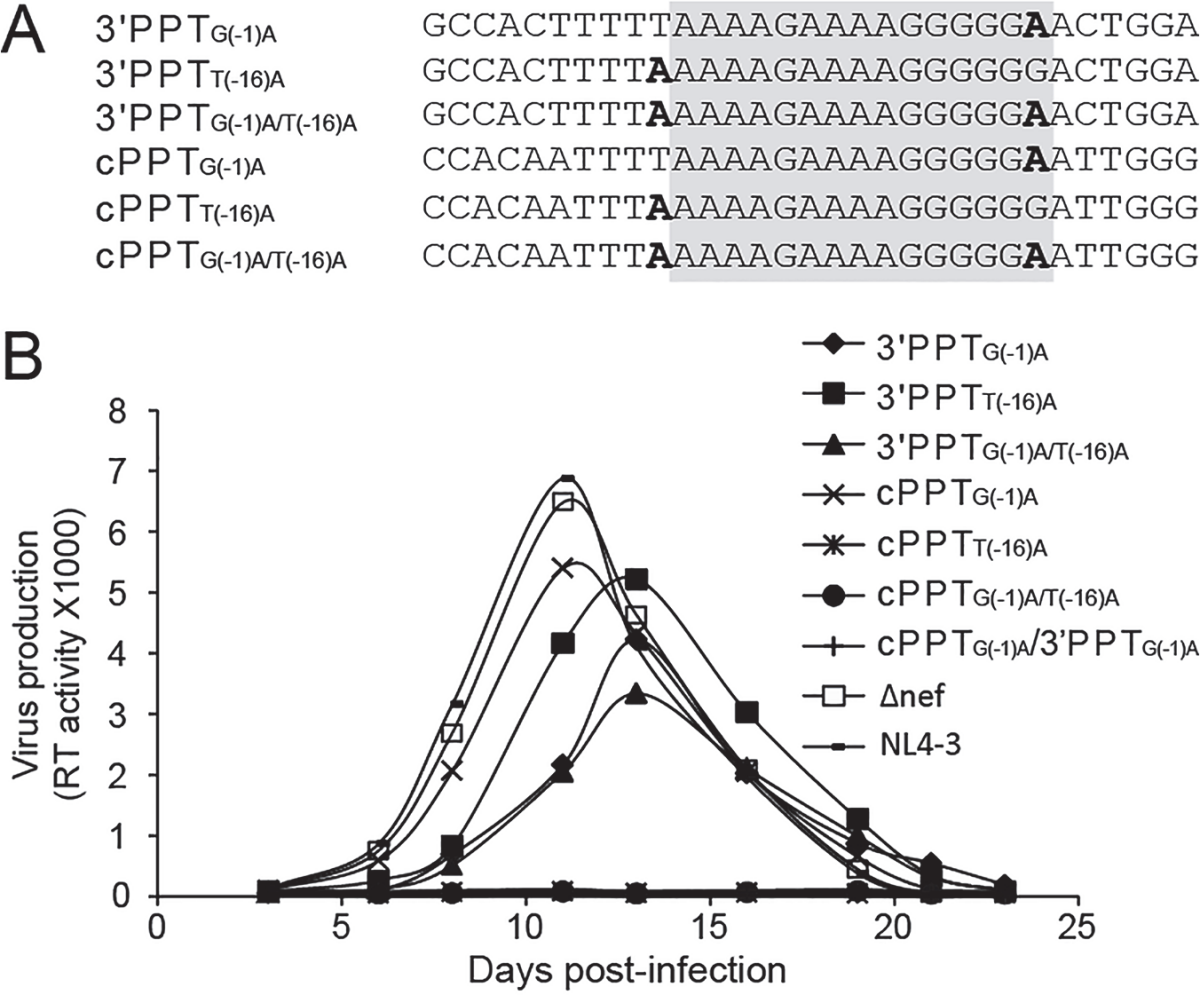
Replication of 3′PPT and cPPT mutant viruses. (A) Introduction of single and double mutations into the 3′PPT or cPPT region in an NL4-3 backbone through yeast based/HIV-1 cloning technology [33]; (B) Replication of various PPT mutants, as well as Δnef mutant (with premature stop codon in nef but outside of PPT region) in U87.CD4.CXCR4 cells. The input viruses were normalized according to RT activity.

In the subsequent analysis of virus replication, all three 3′PPT mutants (3′PPT_G(-1)A_, 3′PPT_T(-16)A_ and 3′PPT_G(-1)A/T(-16)A_), as well as the cPPT_G(-1)A_ mutant propagated in U87.CD4.CXCR4 cells, while the remaining three mutants (cPPT_T(-16)A_, cPPT_G(-1)A/T(-16)A_ and cPPT_G(-1)A_/3′PPT_G(-1)A_ did not (Fig 3B). Two of the non-replicating viruses were engineered to contain a T(-16)A mutation within the cPPT, and this mutation would be expected to cause a TTT-to-TTA codon change and corresponding F185L substitution within the amino acid sequence of integrase. F185 is located at the dimer interface of the enzyme and is critical for integrase strand transfer activity [40]. Moreover, introducing an F185K solubility enhancing mutation into the integrase gene of HIV has been shown to severely impair, and in some cases completely prevent virus replication [41]. Thus, it is most likely that the replication defects observed in cPPT_T(-16)A_ and cPPT_G(-1)A/T(-16)A_ virus are caused by a defect in integrase rather than a non-functional cPPT. This explanation is strengthened by the observation that introducing the equivalent T(-16)A substitution within the 3′PPT did not significantly impact replication. Moreover, virus containing only a G(-1)A substitution within the cPPT (cPPT _G(-1)A_) retained the capacity to replicate, which is consistent with this substitution producing a synonymous change within the integrase coding sequence. The remaining non-replicating mutant, cPPT_G(-1)A_/3′PPT_G(-1)A_, was the only variant containing a nucleotide substitution in both the central and 3′PPTs. This virus failed to replicate possibly because the fitness costs imposed by mutating both plus-strand primers simultaneously were too extreme to overcome. Please note that all of the propagated mutant viruses were sequenced and confirmed there was no reversion of mutation in the corresponding PPT region.

It is also important to note that introducing G(-1)A and/or T(-16)A substitutions into the 3′PPT results in codon changes in the Nef open reading frame; specifically, G(-1)A produces a GGA (G) to GAA (E) change at position 96, while T(-16)A introduced a stop codon at position 91 [TTA (L) to TAA (stop)]. However, it appears unlikely that mutating or truncating the nef protein has a significant effect on virus production in this assay, since the Δnef construct, which contains a stop codon in the nef open reading frame upstream from the 3′PPT, replicates at levels comparable to wild type NL4-3.

### Sensitivity of HIV-1 3′PPT and cPPT mutant clones to siRNA inhibition

**Fig 4:**
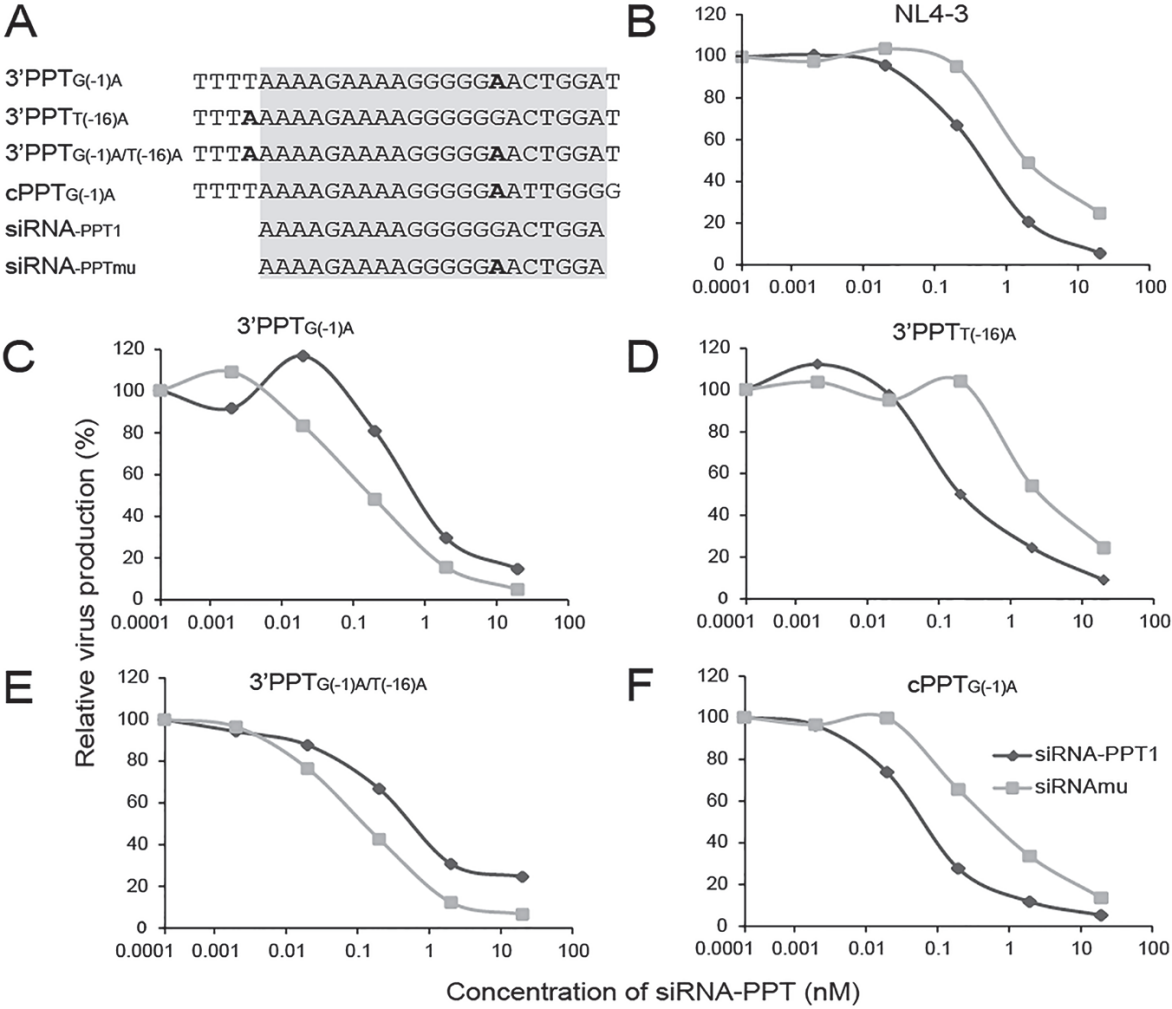
Sensitivity of 3′PPT and cPPT mutant viruses to siRNA-PPT1 and siRNA-PPTmu. (A) PPT and flanking sequences of infectious 3′PPT and cPPT mutants together with siRNA-PPTmu. (C)-(F) Sensitivities of infectious PPT mutants to siRNA-PPT1 and siRNA-PPTmu: (C) 3′PPT_G(-1)A_, (D) 3′PPT_T(-16)A_ (E) 3′PPT_G(-1)A/T(-16)A_ and (F) cPPT _G(-1)A_. (B) Sensitivity of wild-type NL4-3 to siRNA-PPT1 and siRNAmu. Data was based on three independent experiments.

HIV-1 NL4-3 viruses containing different mutations in the 3′PPT or cPPT region were tested for their sensitivity to either siRNA-PPT1 or siRNA-PPTmu in U87.CD4.CXCR4 cells. The latter siRNA is a variant of siRNA-PPT1 designed to target 3′PPTs containing a G(-1)A substitution (A). Based on IC50 values, NL4-3 harboring either the 3′PPT G(-1)A mutation (3′PPT_G(-1)A_) or the tandem G(-1)A and T(-16)A substitutions (3′PPT_G(-1)A/T(-16)A_) were approximately 5-fold more resistant to siRNA-PPT1 than to siRNA-PPTmu (Fig 4C and 4E). In contrast, 3′PPT_T(-16)A_, the only mutant variant tested that does not contain a G(-1)A substitution, was more sensitive to siRNA-PPT1 than to siRNA-PPTmu (Fig 4D). Although the overall levels of resistance are not high in any case, these data indicate that the relative sensitivity of viruses to siRNA-PPT1 and siRNA-PPTmu is effectively determined by whether matching nucleotides are present at the 3′PPT -1 position in both the siRNA and the mutant virus. The identity of the nucleotide at position -16 of the 3′PPT region, which lies outside of the sequence targeted by either siRNA, does not affect siRNA sensitivity. Please note that the siRNA in this study was designed to specifically target 3’PPT, but not cPPT because siRNA-PPT1 also contained sequence outside of PPT region. Therefore, the sensitivity of 3’PPT_G(-1)A_ to siRNA-PPT1mu was lower than the unmutated siRNA-PPT1, but the cPPT_G(-1)A_ had the same sensitivity as it still has the wild-typed 3’PPT.

Finally, we observed that the relative sensitivity of the cPPT_G(-1)A_ variant to siRNA-PPT1 and siRNA-PPTmu (Fig 4F) was similar to that of 3′PPT_G(-16)A_ (Fig 4D). The sensitivity of the cPPT_G(-1)A_ variant to siRNA PPT1 was also similar to that observed for wild-type NL4-3 (compare Fig 4F and Fig 4B). Taken together, these data confirm that both siRNAs target and exert their effect on the 3′PPT, and that mutation of the cPPT is inconsequential in this regard.

### Impact of 3′PPT and cPPT mutations on HIV-1 replicative fitness

Although the presence of siRNA-PPT1 exerted substantial selection pressure on HIV-1 replication, only one escape mutant emerged among three HIV-1 strains tested, suggesting that the selective pressure to conserve the HIV-1 3′PPT sequence is also quite high. Interestingly, both mutations in the emergent v120-A virus were within or near the 3′PPT, while no substitutions were found within the cPPT. It may be that differences in sequence immediately downstream from the respective PPTs or a difference in RNA secondary structure between the two regions rendered the cPPT resistant to targeting by siRNA-PPT1. Alternatively, changes to the cPPT that might otherwise render the element resistant to siRNA targeting may be poorly tolerated in HIV-1 because of concomitant changes to the integrase coding sequence. To address the effects of the observed PPT sequence changes on viral fitness, the replication competent NL4-3 viruses engineered to contain subsets of these mutations in 3′ and cPPT contexts were subjected to a competitive heteroduplex tracking assay (HTA).

**Fig 5:**
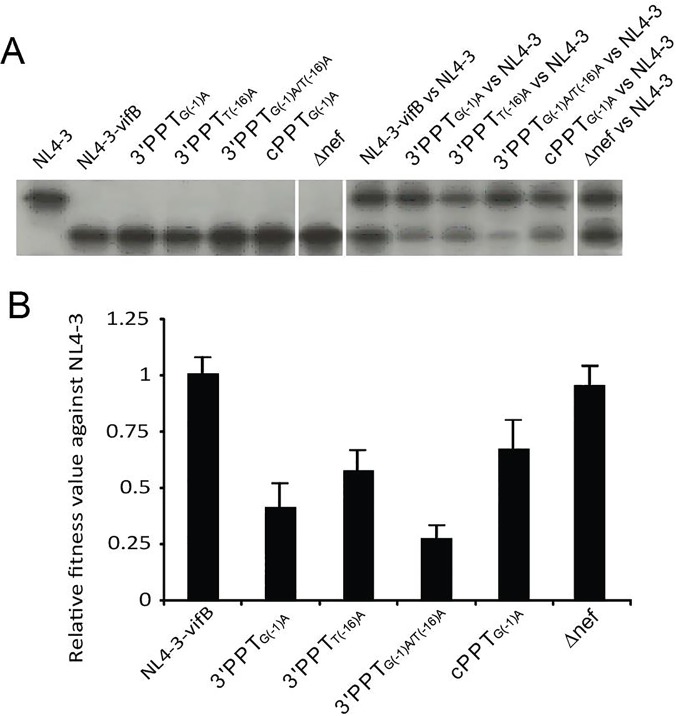
The influence of PPT-region mutations on viral fitness. (A) Example of heteroduplex tracking assay (HTA) gel used to measure the viral fitness of PPT Δnef mutants. Migration of heteroduplexes produced in single or dual/competitive infections are shown on the left and right sides of the panel, respectively. (B) Relative fitness values of PPT and Δnef mutants compared with NL4-3.

Each of the replicating PPT mutant viruses was competed against the parental HIV-1 NL4-3 and showed reduced replicative fitness (Fig 5). 3′PPT_G(-1)A_, despite having only a single nucleotide substitution in the 3′PPT, was found to be significantly less fit than wild-type NL4-3. The mutant containing the equivalent nucleotide substitution in the cPPT (cPPT _G(-1)A_) was also less fit than wild type, although the replication deficiency was not as pronounced. Interestingly, 3′PPT_G(-1)A/T(-16)A_ was significantly less fit than wild-type and any of the replication competent mutants (Fig 5), indicating that in this assay, T(-16)A does not effectively compensate for the reduction in fitness caused by the G(-1)A mutation in the 3′PPT.

One difficulty with interpreting the impact of PPT mutations on viral replicative fitness is determining how much of the effect is due to altered PPT function and how much is caused by altering the coding sequence of the integrase and/or nef protein. For example, as noted previously (Fig 3), introducing a T(-16)A mutation into the cPPT produces a concomitant mutation in the integrase gene, with the combined effect that virus replication is completely abolished. Similarly, introducing nucleotide substitutions into the 3′PPT produces changes in the Nef open reading frame. In the virus production assay (Fig 3), similar peak virus production levels were observed for PPT mutants 3′PPT_G(-1)A_, 3′PPT_T(-16)A_ and 3′PPT_G(-1)A/T(-16)A_ and the truncated nef mutant Δnef, suggesting that altering the nef protein by mutating the Nef open reading frame inside or outside of the PPT sequence had minimal effect on overall virus production. The competitive heteroduplex tracking assay results likewise indicate that truncating nef has little effect on virus fitness under these conditions, and that impaired 3′PPT function, not Nef codon usage, was responsible for the observed decreases in virus fitness (Fig 5).

### Mutations in the PPT region causes improper cleavage of PPT RNA-DNA hybrids

**Fig 6:**
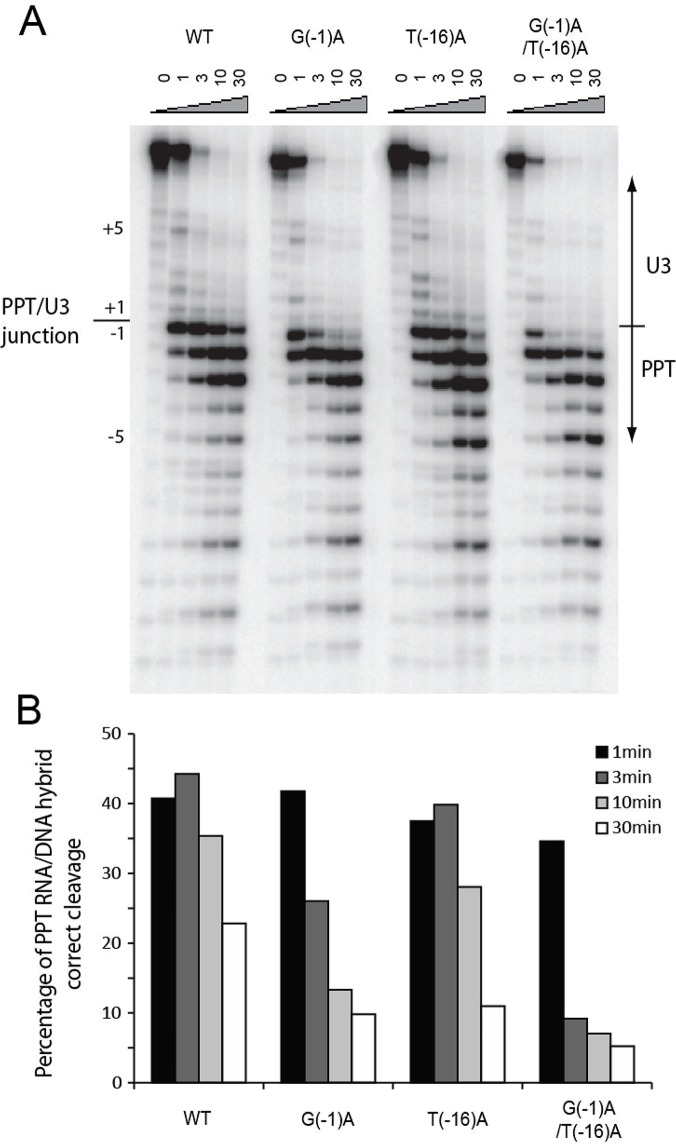
In vitro PPT RNA/DNA hybrids cleavage assay. (A) PPT RNA-DNA hybrid substrates (wild type, G(-1)A, T(-16)A, and G(-1)A/T(-16)A double mutants) were subjected to RNase H-mediated cleavage by HIV-1 RT in the absence of nucleotides. Reactions were terminated at 0, 1, 3, 10, or 30 min; (B) Quantitative analysis of PPT RNA-DNA hybrid cleavage.

Short 29nt/39nt RNA-DNA hybrids were synthesized to contain wild type or mutant 3′PPTs and flanking NL4-3 sequences. Substrates were then subjected to RNase H-mediated hydrolysis by HIV-1 RT in the absence of dNTPs to determine the effects of siRNA-PPT1 resistance mutations on RNase H cleavage patterns. As expected, initial cleavage of the WT sequence occurs predominantly at the PPT-U3 junction (the -1/+1 nt position) as indicated in the autoradiograph and associated bar graph (Fig 6A and 6B, respectively). Some cleavage internal to the wild type PPT is also observed as the reaction progresses (especially at -2/-1 and -3/-2), although this is in part because RT cannot immediately utilize the nascent plus-strand primer due to the absence of dNTPs.

By comparison, all of the mutant PPTs tested are internally cleaved to a greater extent than the wild-type, although this effect is most apparent in mutants containing the G(-1)A substitution. Cleavage of the G(-1)A PPTs at the -2/-1 and -3/-2 junctions is especially pronounced, a result that correlates with a shift in the G-A junction produced by nucleotide substitution at position -1 (i.e., wild-type 5′-GGGGGG^A at the 3′ end of the PPT becomes 5′-GGGGG^AA in the mutants). The effect of this and other internal cleavage events is that the plus-strand primer is rapidly reduced to a length that is sub-optimal for RT binding, thereby reducing the efficiency of plus-strand initiation. Specifically, after 3 minutes, 44.3% of the wild-type 3′PPT is correctly cleaved at the plus-strand primer 3′ terminus, compared with ~26% and ~9.2% for mutant G(-1)A and G(-1)A/T(-16)A, respectively (Fig 6B). These accelerated rates of internal cleavage are in contrast with processing of the T(-16)A PPT variant, where nearly 40% of the correctly cleaved plus-strand primer remains after 3 min.

Taken together, these results indicate that each of the mutant PPTs, especially those containing the G(-1)A nucleotide substitution, are aberrantly processed by HIV-1 RT-associated RNase H. This in turn is likely to affect the efficiencies of plus-strand priming, primer removal and perhaps even integration. Unfortunately, the effects of the G(-1)A and T(-16)A mutations on cleavage at the PPT 5′ terminus cannot be addressed in this assay, and therefore remain unclear. It is possible that the T(-16)A mutation, which appears not to affect siRNA resistance, was present in the 3′PPT of the original v120-A escape variant because it effectively extends the mutant plus-strand primer to the wild type length; i.e. from 5′-UUU^UUAAAAGAAAAGGGGGG^A (17-nt) for the wild type to 5′-UU^UUAAAAAGAAAAGGGGG^AA (17 nt) for the mutant. Alternatively, the T(-16)A mutation may have been produced by slippage during reverse transcription [42] and was retained only because the selection pressure against it was negligible. Regardless, of the two mutations identified in the v120-A escape variant, T(-16)A has the lesser effect on both RNase H processing of the HIV-1 PPT and on viral fitness.

## Discussion

The HIV-1 polypurine tracts are highly conserved genetic elements required for initiation of plus-strand DNA synthesis and intriguing candidates for siRNA targeting. The data presented here provide insight into (i) whether HIV replication can be effectively suppressed in this manner, (ii) which siRNAs most effectively target the PPT, (iii) the degree to which HIV-1 PPTs are genetically flexible and (iv) what nucleotide changes are observed in HIV-1 escape mutants.

Our study demonstrated that siRNA targeting HIV-1 3’PPT region could effectively inhibit virus replication. However, it is not immediately clear why, of the three siRNAs tested, only siRNA-PPT1 was consistently effective in suppressing HIV-1 replication. The explanation for this may lie in the idiosyncrasies of RISC target recognition or perhaps in the unique structure of duplexes containing 3′PPT RNA. Standard synthetic siRNAs are comprised of 19 nt RNA duplexes with 2 nt 5′ overhangs on either side [43]. The complementary 21 nt oligoribonucleotides that constitute these siRNAs are functionally asymmetrical, as it is hybridization of the guide strand to its target that is essential for ultimate degradation of the mRNA. In general, the RNA strand with greater base-pairing thermostability toward the 5′ terminus is preferentially selected as a guide strand in the RISC [44, 45]. However, of the siRNAs utilized in the present study, siRNA-PPT1 does not seem to have an advantage in this regard. Instead, it is possible that the full length PPT sequence (which is only included in siRNA-PPT1) possesses unusual structural features that promote formation of RISC and increase RISC cleavage efficiency just as they direct RT-associated RNase H to generate the viral plus-strand primers. The fact that RNases H and the RISC protein argonaute are in the same gene family makes this possibility even more intriguing. On the other hand, as showed in Fig 4, interestingly, the inhibition efficiency of siRNA inhibiting HIV-1 replication was not significantly decreased when siRNA-PPT1 was mutated with one nucleotide mismatching to its target sequence, which is possibly due to the mutation not sitting in the critical position[46], but may again suggest the above mentioned possibility.

The notion that duplexes containing the intact PPT are in some way exceptional is supported by predictions that hybrid duplexes containing an intact Mo-MLV PPT are more stable than those containing other Mo-MLV genomic RNA sequences of the same length [47]. Alternatively, perhaps hybridization of oligonucleotides comprised of mixed PPT/non-PPT sequence (like siRNA-PPT3) is inherently disfavored. The HIV-1 genomic sequence itself may lend support to this hypothesis. Specifically, there are numerous purine rich sequences throughout the genome that share partial homology with the central and 3′PPTs (e.g., 5′-AAACACAGTGGGGGGACA, 5′-AAAAAACATCAGAAAGAA, etc.) yet do not effectively serve as plus-strand primers. It is possible that the structural and thermodynamic properties that make these sequences unsuitable for plus-strand priming also render PPT targeting with siRNA-PPT2 and/or-PPT3 unfavorable.

The lone escape mutant identified by siRNA targeting of the 3′PPT contained two nucleotide substitutions, G(-1)A and T(-16)A, located at opposite ends of the conserved purine rich element. Together, these mutations conferred both limited resistance to siRNA-PPT1 and reduced fitness in the context of the NL4-3 strain of HIV-1. Interestingly, nucleotide substitution between positions -1 and -16 of the 3′PPT were not observed, suggesting that (i) such mutations would not confer resistance to siRNA or (ii) more significant changes to the PPT could not be tolerated by the virus. We suspect the latter conclusion is more likely, as HIV-1 RT and the HIV-1 PPTs are likely to have co-evolved into a tight functional symbiosis. A comparison of PPT sequences and RT structures among retroviruses tends to support this notion; i.e., differences in PPT sequence between two retroviruses are usually matched by corresponding differences in RT structure suggesting the existence of co-evolution between RT and PPT sequence [4].

As discussed previously, placing the T(-16)A mutation in the context of the cPPT completely abolished virus replication, most likely because this nucleotide substitution changes the integrase coding sequence to produce a defective mutant enzyme. Viruses containing any of the other PPT mutations were viable, despite the fact that introducing T(-16)A or G(-1)A mutations into the 3′PPT either truncates or introduces a point mutation into the *nef* protein, respectively. Even though nef is important for disease progression and is considered a pathogenic factor in primate lentiviridae [48], it is not essential for viral replication [49]. Because the effects of disrupting nef function under these conditions appear to be negligible, we conclude that the reduced fitness observed for virus containing T(-16)A, G(-1)A or both mutations in the 3′PPT region is due mainly to aberrant 3′PPT processing.

Precise generation and removal of plus-strand primers from the 3′ and central PPTs has previously been shown to be crucial for HIV-1 replication [2, 14, 50]. While most RNase H-mediated hydrolysis can be imprecise without impairing synthesis of pre-integrative viral DNA, the cleavage events that generate and remove the plus-strand primers must be specific in order to produce linear DNAs that are appropriate substrates for integration. All RNase H cleavage is catalyzed by HIV-1 RT, a p66/p51 heterodimer housing DNA polymerase and RNase H active sites spaced ~18 bp apart relative to duplex nucleic acid substrates. Plus-strand primer processing presents a unique problem for RT in that the enzyme must first bind the PPT/DNA hybrid in an orientation that positions the RNase H domain for cleavage at the PPT 3′ terminus, then re-bind in the opposite orientation to initiate DNA synthesis from the nascent plus-strand primer. This “flipping” on the substrate has been observed in single molecule FRET studies of HIV-1 RT-PPT/DNA complexes, where it appears to occur more frequently than on generic RNA/DNA hybrids [51, 52]. PPTs must also be refractory to internal cleavage, since a smaller RNA fragment would likely not remain hybridized to minus-strand DNA long enough to promote priming, nor could RT efficiently bind to or initiate DNA synthesis from a truncated PPT primer.

The structural determinants that dictate RT binding orientation, direct proper RNase H cleavage, prevent internal PPT cleavage and promote plus-strand priming have been extensively studied yet remain poorly understood [4, 25, 53]. Numerous contact points exist between primer grip/RNase H primer grip residues in HIV-1 RT and the DNA strand of an RNA/DNA hybrid, some of which have been suggested to play a role in specific recognition of the PPT/DNA hybrid [54–56]. On the nucleic acid side, previous work has demonstrated that introducing G-to-A substitutions at positions -2 and/or -4 in the HIV-1 PPT resulted in enhanced internal cleavage, while a contiguous stretch of 3′ terminal Gs has been shown to promote priming of DNA synthesis in both RNA/DNA and DNA/DNA contexts [25, 57]. The role of the 5′-terminal A-tracts in HIV-1 PPT function is less apparent, as only modest effects on PPT processing are observed upon site-directed mutagenesis of individual A-tract nucleotides [25, 53]. However, complete removal of one or both A-tracts greatly reduces the efficiency of plus-strand primer generation and utilization [25]. Some evidence also suggests that the U-A junction at the PPT 5′ terminus is important for preventing slippage during reverse transcription of HIV-1 RNA, as demonstrated in SIV mutants in which the U-tract immediately upstream from the 3′PPT had been deleted [42].

Introducing the G(-1)A mutation into a synthetic PPT/DNA hybrid increases the rate of internal PPT cleavage, particularly at the -2/-1 position. This effect is not negated in the G(-1)A/T(-16)A tandem mutation, suggesting that, in the context of an in vitro RNase H assay, the second mutation is relatively inconsequential. Internal cleavage of the PPT at any position is likely to reduce the efficiency of plus-strand priming both because a shorter RNA primer will not remain stably associated with the minus-strand DNA complement and because fewer contact sites will be available for RT binding (and initiation events will therefore be less frequent). In addition, HIV-1 pre-integrative DNA would be expected to be one-or-more nucleotides longer, depending upon whether the truncated 3′PPT is successfully removed, which is likely to affect integration.

Pre-integrative DNA 3′-processing exposes the 3′ hydroxyl in the integrase/dsDNA complex for nucleophilic attack on the host genomic DNA—an event known as strand transfer. Retroviral integrase attachment sites (*att*) are found at the extreme U3 and U5 ends of the linear proviral genome, as long as the pre-integrative DNA is correctly processed [58–61]. The *att* is comprised of at least 7 and as many as 20 bp starting from the highly conserved 3′-ACTG-5′ at the terminus of U3. Extensive mutational analyses of this relatively well-conserved U3 terminus revealed that nucleotide substitutions in numerous positions are important for IN recognition [59, 61–63]. However, to our knowledge, there have been no reports on the impact of U3 terminal extensions on integrase activity and specificity. It is quite possible that a single base pair extension on the end of U3, or a more significant extension in the event the plus-strand primer is not properly removed, could reduce IN binding and dinucleotide cleavage. Dicker et al. [64] compared the relative binding affinity of inhibitors and various mutant *att* U3 sequences for integrase. Because these ligands/inhibitors bind integrase cooperatively, mutations within the terminal four base pairs of *att* U3 (or *att* U5) not only reduce the affinity of integrase for its nucleic acid substrates but also, indirectly, for the inhibitors. Interestingly, our NL4-3 virus with G(-1)A or the G(-1)A/T(-16)A substitutions in the 3′PPT were slightly less sensitive to the integrase inhibitor Raltegravir than the wild-type virus (data not shown). These findings suggest that a 1 nt U3 extension may exist in viral DNA and that this extension may reduce integrase activity.

## Supporting Information

S1 Fig Generation of various 3′PPT and cPPT mutated molecular clones and corresponding viruses using a yeast based/HIV-1 cloning system. (A) Construction of a 3′PPT mutant, pREC_nfl_HIV-1_3′PPT_G(-1)A_ using a yeast based/HIV-1 cloning technology [33]; (B) Generation of infectious virus, 3′PPT_G(-1)A_ through co-transfection of pREC_nfl_HIV-1_3′PPT-a and the complementary vector pCMV_cpltRU5_gag/tag; (C) Complementation of viral reverse transcription by jumping between R regions in the two viral subgenomic RNAs.
(TIF)

S1 Dataset Dataset for Fig 1, Fig 2, Fig 3, Fig 4, Fig 5, and Fig 6. (XLS) (XLS)
