# Supplementary figures and images for: SiRNA-Induced Mutation in HIV-1 Polypurine Tract Region and Its Influence on Viral Fitness
Source: PLoS One. 2015 Apr 10;10(4):e0122953. doi: 10.1371/journal.pone.0122953 (PMC4393142; doi:10.1371/journal.pone.0122953)

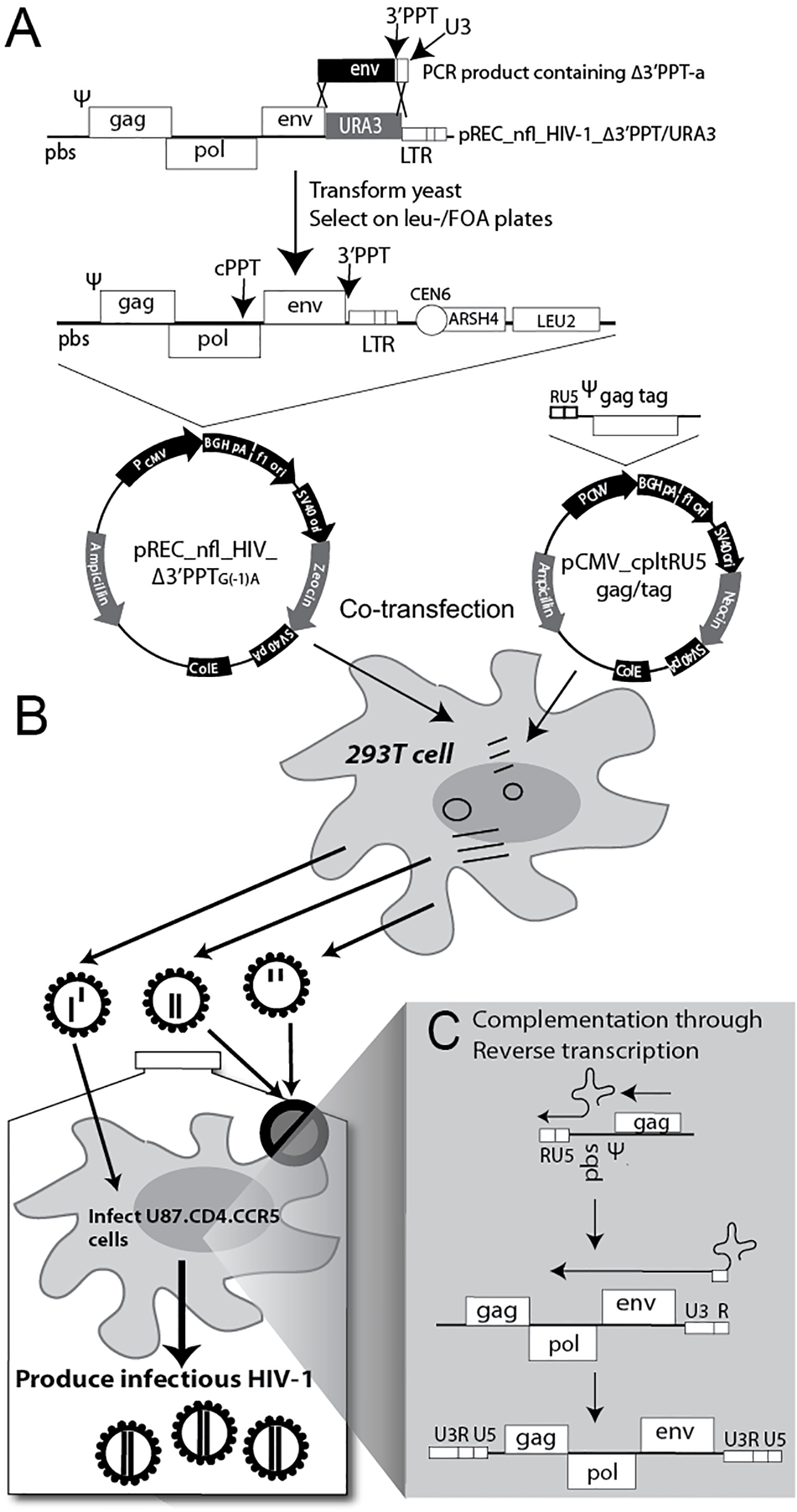

Supplement: S1 Fig — (A) Construction of a 3′PPT mutant, pREC_nfl_HIV-1_3′PPTG(-1)A using a yeast based/HIV-1 cloning technology [33]; (B) Generation of infectious virus, 3′PPTG(-1)A through co-transfection of pREC_nfl_HIV-1_3′PPT-a and the complementary vector pCMV_cpltRU5_gag/tag; (C) Complementation of viral reverse transcription by jumping between R regions in the two viral subgenomic RNAs. (TIF) [file pone.0122953.s001.tif]
